# Role of thromboelastography in the management of haemorrhage: an observational analysis

**DOI:** 10.1186/cc14423

**Published:** 2015-03-16

**Authors:** C Ward, J Aron, A Gibbon, J Ball

**Affiliations:** 1 St George's Hospital, London, UK

## Introduction

The aim of this analysis is to explore and evaluate the role of thromboelastography (TEG) in aiding the management of patients admitted with haemorrhage to the ICU. TEG has been shown to rationalise and reduce transfusion requirements [1] but the precise role of TEG in the assessment and management of haemostasis in the bleeding patient is uncertain and has not been previously demonstrated.

## Methods

A prospective audit of TEG analyses performed on patients in the general medical and surgical ICU was recorded over a 2-month period. Operators documented the reason for admission, demographic data, indication for TEG, laboratory results, blood gas analysis, TEG results, diagnosis and subsequent action from the TEG result. Only patients who had been admitted with haemorrhage were included in this analysis.

## Results

Seventy-eight audit sheets were completed, of which 31 identified haemorrhage as the reason for admission. The mean age was 59.3 (range 21 to 90) and the mean APACHE II score was 18.23 (range 11 to 37). The main indications for TEG analysis included coagulopathy (64%) and ongoing haemorrhage (45%). As a result of performing TEG analysis, 23 (74%) patients had a documented change in their management. Ten patients did not require any further administration of blood products, which they would have received based on conventional laboratory results. The information gained from TEG also resulted in the omission of anticoagulation in three patients, and with a further two patients anticoagulation increased.

## Conclusion

TEG aids prompt rationalisation of blood products and titration of anticoagulation in the bleeding patient. TEG identifies a number of patients who required administration of platelets and other procoagulants which would not have been identified by conventional methods. Several patients would have also received inappropriate transfusions which has both cost and resource implications, alongside the potential adverse effects on patients. We recognise that further research is needed to clarify the overall efficacy of TEG in the bleeding patient.
